# Supplementary material for: MetaRibo-Seq measures translation in microbiomes
Source: Nat Commun. 2020 Jun 29;11:3268. doi: 10.1038/s41467-020-17081-z (PMC7324362; doi:10.1038/s41467-020-17081-z)
Supplement: Supplementary file 10 — Supplementary Data 7 [file 41467_2020_17081_MOESM10_ESM.zip › File2/Confidence_VeryHigh_Taxonomy/92857_out.krona.html]

Javascript must be enabled to view this page.

members
magnitude
magnitudeUnassigned
count
unassigned
taxon
rank

92857\_out

4


SRS024663\_contig\_number\_6502
superkingdom
3
1
2

1239
2
phylum

186801
2
class

186802
order
2

species
2

SRS1041092\_contig\_number\_15940SRS144714\_contig\_number\_28717
172733


SRS893373\_contig\_number\_4715
1
